# Detection and Genetic Analysis of Porcine *Bocavirus* in Different Swine Herds in North Central China

**DOI:** 10.1155/2014/947084

**Published:** 2014-02-18

**Authors:** Mengmeng Liu, Yi Li, Dong Sun, Yihe Xia, Jinhai Huang, Lili Guo

**Affiliations:** ^1^School of Chemical Engineering and Technology, Tianjin University, No. 92, Weijin Road, Nankai District, Tianjin 300072, China; ^2^Department of Veterinary Diagnosis and Production Animal Medicine, College of Veterinary Medicine, Iowa State University, Ames, IA 50011, USA; ^3^Tianjin Center of Animal Disease Preventive and Control, Tianjin 300012, China

## Abstract

Porcine *Bocavirus* (PBoV) has been reported to be associated with postweaning multisystemic wasting syndrome and pneumonia in pigs. In this study, a survey was conducted to evaluate the prevalence of PBoV in slaughter pigs, sick pigs, asymptomatic pigs and classical swine fever virus (CSFV) eradication plan herds in five provinces of China (Henan, Liaoning, Shandong, Hebei and Tianjin) by means of PCR targeting NS1 gene of PBoV. Among the total of 403 tissue samples, 11.41% were positive for PBoV. The positive rates of spleen (20.75%) and inguinal lymph node (27.18%) are higher than those of other organs. PCR products of twenty PBoV positive samples from slaughter pigs were sequenced for phylogenetic analysis. The result revealed that PBoV could be divided into 6 groups (PBoV-a~PBoV-f). All PBoV sequenced in this study belong to PBoV-a–PBoV-d with 90.1% to 99% nucleotide identities. Our results exhibited significant genetic diversity of PBoV and suggested a complex prevalence of PBoV in Chinese swine herds. Whether this diversity of PBoV has a significance to pig production or even public health remains to be further studied.

## 1. Introduction

Parvoviruses, members of the family Parvoviridae, are able to cause a broad spectrum of diseases in animals [1]. The family Parvoviridae is divided into two subfamilies: Densovirinae and Parvovirinae. In the subfamily Parvovirinae, genus *Bocavirus* is uniquely different from other four genera, *Amdovirus*, *Dependovirus*, *Erythrovirus*, and *Parvovirus*, in the genome components (http://ictvonline.org/). *Bocavirus* contains a single-stranded, 4–6 kilobases (kb) long, autonomously replicating DNA genome with terminal palindromic sequences [2–5]. In total, 4 open reading frames (ORFs) have been identified: ORF1 at the 5′ end encodes nonstructural protein NS1; ORF2 which is in the middle of the genome encodes nonstructural protein NP1, a unique *Bocavirus* characteristic structure that is absent in most other members of Parvoviridae [5]; overlapping ORF3 and ORF4 encodes structural proteins VP1 and VP2, respectively [5].

*Bocavirus* was discovered in both humans and animals [6, 7]. In 2009, a novel boca-like virus was discovered in Swedish postweaning multisystemic wasting syndrome (PMWS) suffering pigs using random amplification and large-scale sequencing techniques [8–10]. PBoV has been detected in PMWS-affected swine herds, suggesting that it might also play a role in the pathogenesis of PMWS [8, 11]. The PBoV grouping is confusing in different reports [6, 12] when different regions of genome (genome, NS1, VP1, or NP1) were used in the phylogenetic analysis and thus make the differentiating of novel PBoVs difficult. Porcine *Bocavirus* (PBoV1) based on partial VP1/2 genes was first discovered in PMWS affected pigs in Sweden [10], and then the complete genome of PBoV types 1 and 2 was characterized and reported in 2010 [12, 13]. Two novel PBoV types, 3 and 4, were discovered in Hong Kong based on the phylogenetic analysis of selected VP1 gene fragments [14]. The complete genome of PBoV type 5 was discovered and identified in piglet stool samples from a farm where piglets are experiencing clinical diarrhea [15]. Based on nonstructural (NS) gene, according to International Committee of Taxonomy of Viruses, PBoV can be classified into more than 5 genotypes with less than 95% sequence identity (http://ictvonline.org/) among different genotypes [6, 15].

The prevalence of *Bocavirus* in human has been reported as ranging from 1.5% to 24.6% in different human populations around the world [7]. Moreover, increasing evidences are emerging to support the role of *Bocavirus* as an etiologic agent in human lower respiratory tract infection [12]. Due to the close relationship between human and pigs, the possibility that human might be infected by PBoV cannot be completely ruled out, and studies on PBoV could potentially benefit public health. In China, discovery of PBoV has been intensively reported. It is necessary to understand the PBoV epidemic status and gene group distribution based on the identical NS1 gene standard. In this study, a survey on the prevalence of PBoV in different parts of China was conducted by PCR detection on collected pig tissue samples with NS1 gene specific primers. Partial PBoV NS1 genes of 20 positive samples were sequenced and compared with reference sequences. Our observations could provide information for understanding the circulation and variety of PBoV in different herds or regions of China and also for evaluating the role of PBoV in public health issue.

## 2. Materials and Methods

### 2.1. Sample Collection

A total of 403 pig samples from individual animals with variant tissue origins including inguinal lymph node, submandibular lymph node, lung, kidney, liver, and spleen were collected from five provinces in China from 2006 to 2012. Among the 403 samples, 183 were from slaughter pigs (100 from 2 farms in Henan, 43 from a farm in Shandong, and 40 from a farm in Liaoning), 70 were from sick pigs with clinical signs, including diarrhea, mild cough,or low fever in Hebei, 150 tonsils were collected from 60-day-old replacement gilt (130 from classic swine fever virus eradication plan performing herds, which are CSFV-free and 20 samples from noneradication plan performing gilt herds) in Tianjin breeding pig farm. Detailed sample information was summarized in Table 1. All collected samples were stored at −80°C until test was performed.

### 2.2. DNA Extraction

Each porcine tissue sample (100 mg) was frozen in liquid nitrogen, grounded with a homogenizer, and suspended in 500 *μ*L PBS (0.01 mol/L, pH7.2). Approximately 300 *μ*L homogenate was used for DNA extraction with conventional phenol-chloroform extraction method. Briefly, the homogenate was mixed with 300 *μ*L lysis buffer (containing 10 mM Tris-Cl, 1 mM EDTA, and 0.1% SDS) and 10 *μ*L proteinase K (10 mg/mL) and digested for 2 hours at 60°C. The sample was then centrifuged for 10 min at 12,000 ×g. The aqueous upper phase was transferred to a new tube with 600 *μ*L Tris phenol added, then mixed by inverting for 3 min, and centrifuged for 10 min at 12,000 ×g. The supernatant was transferred to a new tube mixed with the equal volume of phenol : chloroform : isoamyl alcohol (25 : 24 : 1) mixture and then centrifuged for 10 min at 12,000 ×g. The aqueous upper phase containing DNA was precipitated with 1/10 volume of sodium acetate (3 mol/L, pH5.2) and 2 volumes of precooled ethanol at −20°C for 2 hours. After centrifugation for 10 min at 12,000 ×g, the DNA was washed with 70% ethanol, then dried naturally, and dissolved with TE buffer (RNase included). The extracted DNA was stored in −20°C for further use.

### 2.3. PBoV Detection

To enlarge the detection probability of different PBoV isolates, primers (PBoV-F: 5′-ACAGGCAGCCGATCACTCACTAT-3′ and PBoV-R: 5′-CTCGTTCCTCCCATCAGACACTT-3′) were designed based on the conservative regions of NS1 gene. Each DNA extract (20 ng/*μ*L, approximately) was amplified with PCR mixture containing 0.5 *μ*L of 10 *μ*M PBoV-F, 0.5 *μ*L of 10 *μ*M PBoV-R, 5 *μ*L of 10× PCR buffer, 0.1 U Taq DNA Polymerase, 1 *μ*L of 0.4 mM each dNTPs, and 1.5 *μ*L of DNA. DNAase-RNAase free water was added to make a total volume of 20 *μ*L. Amplification reaction consisted of initial denaturation at 94°C for 6 min, 35 cycles of denaturation at 94°C for 20 sec, annealing at 52°C for 30 sec, extension at 68°C for 55 sec, and final extension after cycling at 72°C for 5 min. The PCR products were separated by electrophoresis on 1% agarose gel, and a band at the 680 bp position confirmed the presence of PBoV.

### 2.4. Sequencing and Phylogenetic Analysis

PCR products of twenty PBoV positive samples were purified using a commercial PCR purification kit (Omega Bio-Tek, Inc, USA) and sequenced at the Beijing Genomic Analysis Center (Beijing). The sequences were deposited in GenBank and then were screened for sequence similarities using BLASTn against the nr database in GenBank. In addition, fifteen PBoV sequences from UK (GenBank accession numberS JF512472, JF512473), USA (JF713714, JF713715), Hong Kong (JF429834, JF429835, and JF429836), and China (JN621325, JN831651, JN681175, HM053693, HM053694, HQ291308, HQ291309, and HQ223038), five human *Bocavirus* isolates (JX257046, NC 012729, NC 012564, JN632511, and JX887481), one Feline *Bocavirus* (NC 017823), one California sea lion *Bocavirus* 2 (JN420366), one Canine *Bocavirus* (JQ692588), and one Myotis *Bocavirus* 1 (JQ814850) were downloaded from GenBank and used for phylogenetic analyses. For amino acid analysis, sequences were manually adjusted for the correct reading frame.

Phylogenetic trees were generated using the neighbor joining (NJ) method with bootstrap of 1,000 replicates using MEGA 5.1 software (http://www.megasoftware.net). Percent bootstrap support was indicated at each node. GenBank accession number was indicated at each branch.

## 3. Results

### 3.1. Prevalence of PBoV among Different Pig Herds in China

Overall of the 403 samples tested, 46 (11.41%) were found to be positive for PBoV by PCR (Table 1). The prevalence of PBoV in inguinal lymph node (27.18%, 28/103) and spleen (20.75%, 11/53) was significantly higher than that in submandibular lymph node (6.25%, 5/80) and tonsil (1.30%, 2/130). No PBoV was tested in lung (0%, 0/8), kidney (0%, 0/3), and liver (0%, 0/6) tissues.

Prevalence differences were observed existing in slaughter swine samples among different regions of China. Samples collected from Shandong showed a higher PBoV prevalence (41.86%, 18/43) than those of Henan (11%, 11/100) and Liaoning (10%, 4/40).

### 3.2. Phylogenetic Analysis of PBoV

PCR products of 20 PBoV positive samples were selected and sequenced. The sequences were deposited in GenBank (accession numbers JX885585, JX944649–JX944667).

**Figure 1:**
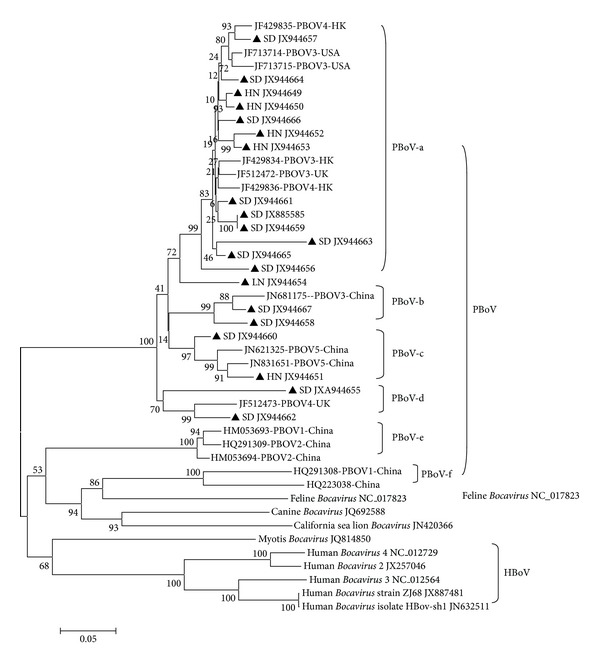
Phylogenetic tree constructed based on partial NS1 sequences of reference PBoV and PBoV from Chinese slaughter pigs. Slaughter pig sequences are labeled with ▲ and their corresponding accession numbers. The phylogenetic tree was constructed by MEGA ver. 5.1 with neighbor-joining method using 1,000 bootstrap replicates.

Phylogenetic analysis was performed based on a 680 bp fragment of NS1 gene using the 20 samples in this study and 24 representative reference strains from different hosts and locations over the world (Figure 1). Comparative sequence analysis of NS1 genes from PBoV, human *Bocavirus*, and other animal *Bocaviruses* showed distinguishable gene cluster among all those *Bocavirus* isolates. All PBoV NS1 sequences had 71.4% to 99% nucleotide identity with human *Bocavirus* and other animal *Bocaviruses*.

Human *Bocavirus* and animal *Bocavirus* except for PBoV strains had less than 64% identity and formed several separate clades. Feline *Bocavirus* NC017823 NS1 gene had 74.6%–78.3% identity with HQ223038 and HQ291308 strains (PBoV-f group) which were named as PBoV1 in previously report [6]. The phylogenetic effect between PBoV and feline *Bocavirus* should be further evaluated. One canine *Bocavirus* sequence JQ692598 and California sea lion sequence JN420368 had a 69% identity and were grouped to one clade (Figure 1). All 5 human *Bocaviruses* can be divided into different subclades and are grouped separately from porcine *Bocavirus* and other animal *Bocaviruses*. From 88.5% to 92% identity within different human *Bocaviruses* sequences and 78.5% identity with Myotis *Bocavirus* JQ814850 sequences. This preliminary study shows divergence among *Bocavirus* strains [6, 8, 13, 16].

In our study, PBoV was divided into 6 different clades, PBoV-a~PBoV-f. Group PBoV-a to PBoV-f sequences had 93.64%, 91.1%, 94.15%, 90.93%, 96.66%, and 78.3% identities within their group, respectively. Based on differences of PBoV NS1 gene, five gene groups (PBoV1–5) were divided as previous report [6, 15]. PBoV-a sequences have 91.7% to 99% identities with each other as well as about 95% identity with published PBoV3 sequences from Hong Kong (JX429834), USA (JF713715, JF713714) or UK (JF512472), and more, 92%–96% identity with PBoV4 sequences from Hong Kong (JF429835, 429836) [17]. PBoV-b had 2 Shandong isolate sequences (JX944658, JX944667) which gave match with PBoV3 JN681175. PBoV-c had two sequences (JX944660, JX944651) which gave match with PBoV5 (JN621325, JN831651). PBoV-d included 3 sequences (JX944655, JX944662) that gave match with PBoV4 (JF512473). PBoV-e includes two classical PBoV2 (HQ291309, HM053694 strains) and one previously named PBoV1 sequence HM053693. PBoV-f included two PBoV1 strains, HQ291308 and HQ223038, from China. Group PBoVa–d sequences had 89.7% identity while having 81.2% identity with groups PBoV-e and PBoV-f. PBoV-a sequences had 93.64% (from 91.7% to 99%) identity within the group and 87.54% and 88.98% identities with PBoV-e and PBoV-f group sequences, respectively.

In this study, all 20 slaughter pig PBoV NS1 sequences located in PBoVa–d groups mean that all isolated NS1 genes are more relevant to traditional PBoV3–5 group isolates, which did not give match with any PBoV1-2 group reference sequences as previously reports [6, 15]. The result indicated that the diversity genotype PBoV strains existed in China [11, 15].

**Figure 2:**
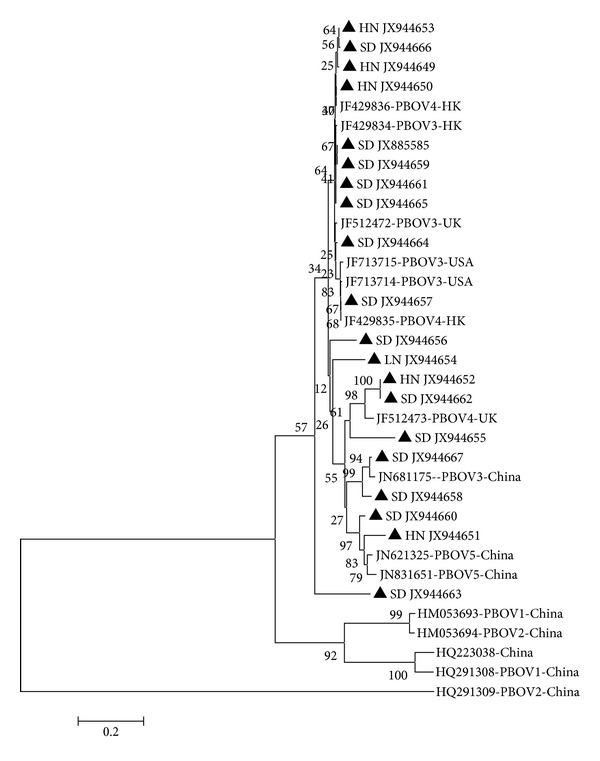
Phylogenetic tree constructed based on partial NS1 amino acid sequences of reference PBoV and PBoV from Chinese slaughter pigs. Slaughter pig sequences are labeled with ▲ and their corresponding accession numbers. The phylogenetic tree was constructed by MEGA ver. 5.1 with neighbor-joining method using 1,000 bootstrap replicates.

About 83.3% amino acid identity of partly NS1 protein sequence was obtained in 20 strains of this study and 15 published porcine *Bocavirus* sequences, including PBoV1–PBoV5 subtypes from China and USA (Figure 2). A similar amino acid sequence alignment result showed that all PBoV strains sequenced in this study are more related to PBoV3, PBoV4, and PBoV5 (90.38% similarity) but distinct from PBoV1 and PBoV2. Basing the clustering on the NS1 gene or amino acid sequences, it was not possible to consistently cluster the strains into similar groups [6]. For example, strains PBoV1-HM053693, PBoV1-HQ291309, and PBoV2-HM053694 in the gene NS1 based phylogenetic tree (Figure 1) were separated by additional PBoV1-HQ291309 in the amino acid NS1 phylogenetic tree (Figure 2). These differences may be due to crossover recombination during the speciation of these viruses as suggested [6, 14].

## 4. Discussion

Our survey tested collected tissue samples from multiple provinces of China. Positive tissue samples were detected from all provinces, implying that PBoV is widely distributed Chinese swine herds. Prevalence rates among provinces are variant. A higher portion of PBoV presence was observed in the samples from Shandong province than those from other provinces. PBoV is distributed differently in tissues. PBoV positive rate detected in inguinal lymph nodes was significantly higher (*P* < 0.05) than other tissue samples (submandibular lymph node, liver, spleen, etc.). Since samples we collected from pigs at different geographical locations are of different tissue origins, the difference of PBoV prevalence in location may be biased by tissue tropism of the virus. Similarly, the difference of PBoV prevalence in tissue could also be biased by geographical location. Further studies are needed to provide more comprehensive insight to this.

In our study, PBoV was divided into 6 different clades, PBoV-a~PBoV-f. Sequence analysis showed that all 20 PBoV sequences were grouped into PBoV-a–d groups and showed a big difference from PBoVe-f group. Our results indicated that the diversity genotype PBoV strains existed in China and the current porcine *Bocaviruses* exhibited significant genetic diversity among different hosts [14].

Currently, the pathogenesis of PBoV remains to be clarified. PBoV can be detected in tissues, serum, and fecal samples from not only clinically normal pigs but also clinically sick pigs suffering from various disease manifestations including porcine circovirus associated disease (PCVAD) and respiratory disease [10, 16–18]. It is well known that porcine parvovirus type 1 is a causative agent of reproductive failure in sows, while the pathogenicity of other types of *Parvoviruses* [6, 11, 19] and members of other genus such as *Bocavirus* or Covirus remain to be determined. Numerous experimental investigations reported that coinfection of PCV2 with other viruses can contribute to the development of the PMWS in pigs [17, 20, 21]. It needs to be confirmed by experiments whether it is the case for PBoV. Yet, the higher prevalence of PBoV detected in PMWS suffering pigs (88%) is compared to pigs without PMWS (46%) [8], and the presence of PBoV in pigs suffering from diarrhea or in weaning pigs with respiratory symptoms suggests that this virus may play a role in the development of PMWS [15] or respiratory diseases [5]. However, the exact role of PBoV, either alone or in combination with other enteric viruses, in causing diarrhea of pigs need to be explored by experiments. In our current study, a higher PBoV prevalence was detected in inguinal lymph node (8/20) than submandibular lymph node (2/20) by comparing 20 lymph node samples from different individuals, suggesting a higher PBoV viral load in inguinal lymph node. In addition, spleen (20.75%) and inguinal lymph node (27.18%) have much higher positive rates compared to other organs, suggesting that they might be the organs where PBoV multiplication takes place. Moreover, different *Bocavirus* sequences can be obtained from different tissues within the same host and that may represent different virus species and viral multiplication [14]. Comparing samples collected from CSFV eradication program performing herds with nonperforming herds in Tianjin breeding farm, only 1 in 130 tonsil samples (0.77%) collected from CSFV eradication program performing herd, were detected as positive, while 1 in 20 tonsil samples (5%) collected from nonperforming herd was positive. The rate is significantly lower (*P* < 0.05) compared to that of eradication program nonperforming herds (5%), which implies that CSFV eradication plan may be of help to decrease coinfection of PBoV. Even though data is less sufficient, we believe that CSFV eradication plan helped to decrease coinfection of PBoV, because generally good farm management could help farms to control diseases better.

*Parvoviruses* have been shown to have mutation rates as high as that of RNA viruses [13, 22]. The phylogenetic analysis confirmed that the recombination event occurred between HBoV1 and HBoV4 and generated HBoV2. HBoV strains were previously considered to have high sequence diversity, very low protein identity, broad intergenotype recombination between HBoV1 and HBoV4, and intragenotype recombination between HBoV2 variants [13, 16]. In addition, recombination analysis showed possible recombination events in VP1 region of PBoV4 strains, suggesting that different strains/variants within the same host could have arisen from recombination, and coinfection of different *Bocavirus* strains could have a high chance of recombination [14, 23]. Many emerging viruses are zoonotic, causing epidemics in humans after overcoming the interspecies barrier through mutation or other genetic events such as recombination. Human *Bocavirus* is associated with lower respiratory tract infection, pneumonia [7]. In addition, the latest report showed that human *Bocavirus*could integrate in the host genome by persisting in some infected tissues in the form of cccDNA and then contribute to the development of some lung and colorectal tumors [24]. Although the 20 PBoV strains sequenced in our study are grouped distinctly with HBoV, PBoV strains grouped in PBoV-f are much more close to feline *Bocavirus*. Moreover, due to the close relationship between human, canine, feline, and pigs, the possibility that human might be infected by PBoV cannot be completely ruled out.

PBoV genomes have been found in slaughter offal (blood, lymph nodes, feces, etc.). Irregular disposal of those waste could disseminate the virus in water and pork. Pork products occupy a large proportion of food consumption in China, and PBoV is a potential human pathogen [7, 18, 25], and thereby PBoV infection in slaughter pigs raises a big concern for food quality and safety and maybe even human welfare and regional trade. Efforts are needed to clarify the potential risks of PBoV to public health. A close attention needs to be put into the monitoring of this agent to ensure a relieved food supply chain.

## Figures and Tables

**Table 1:** Prevalence of PBoV detected by PCR in the pig samples in China.

Origin	Type of tissue or samples	Number positive/number tested (%)	Number sequenced
Kaifeng (Henan)	ILN	9/20 (45.00)	5
	SLN	0/20 (0.00)	
Luohe (Henan)	ILN	2/40 (5.00)	
	SLN	0/20 (0.00)	
Shandong	ILN	15/23 (65.21)	14
	SLN	3/20 (15.00)	
Liaoning	ILN	4/20 (20.00)	1
	SLN	0/20 (0.00)	
Hebei	Kidney	0/3 (0.00)	
	Spleen	11/53 (20.75)	
	Liver	0/6 (0.00)	
	Lung	0/8 (0.00)	
Tianjin	Tonsil (group A)	1/20 (5.00)	
	Tonsil (group B)	1/130 (0.77)	

Total		46/403 (11.41)	20

Note: inguinal lymph node (ILN; *lnn.  inguinales superficiales*); submandibular lymph node (SLN; *lnn.  mandibulares*); tonsil (group A), classical swine fever virus (CSFV) eradication plan herds; tonsil (group B), nonclassical swine fever virus (CSFV) eradication plan herds.
